# Long-term Effect of Smoking on Serum Pepsinogen Values

**DOI:** 10.2188/jea.12.351

**Published:** 2007-11-30

**Authors:** Shogo Kikuchi, Michiko Kurosawa, Tsuguo Sakiyama, Hiroshi Tenjin

**Affiliations:** 1 Department of Public Health, Aichi Medical University School of Medicine.; 2 Department of Epidemiology and Environmental Health, Juntendo University School of Medicine.; 3 Clinic Attached to Kanto-shin-etsu Regional Taxation Bureau.

**Keywords:** long-term effect, smoking, serum pepsinogen, *Helicobacter pylori*

## Abstract

Background: The serum pepsinogen I to II ratio (PG I/II) is related to the risk of stomach cancer. Smoking is an established risk factor for stomach cancer. The effect of smoking on the change in PG I/II over a 7-year span was investigated.

Methods: Data were from 1889 male subjects who underwent phlebotomy in both 1989 and 1996. The subjects were classified into smoking and non-smoking groups: those who continued smoking, and those who never smoked during the span. The subjects were stratified by *Helicobacter pylori* status (negative or positive) and age (20-29 or 30-56 years in 1989), and the change in PG I/II was compared between the smoking and the non-smoking groups.

Results: PG I/II increased less (age adjusted mean ±standard error of the difference was 0.209±0.069, p<0.001) and less frequently in the smoking group (65.8% versus 58.9%, p=0.002), but these differences were not clear among *H. pylori*-positive subjects.

Conclusions: The less frequent increase (i.e. a more frequent decline) in PG I/II may be a long-term effect of smoking, although the effect is not clear under *H. pylori* infection. The decline may be one of the mechanisms through which smoking elevates the risk of stomach cancer.

It is known that serum pepsinogen values are associated with the risk of stomach cancer. A low pepsinogen I to II ratio (PG I/II) indicates high risk of stomach cancer.^1^^-^^3^ On the other hand, many studies have shown that smoking is a risk factor for stomach cancer.^4^ Several cross sectional studies has been carried out to evaluate the relationship between smoking and serum pepsinogen values.^5^^-^^7^ However, so far few studies have assessed the effect of years of exposure to smoking on the change in serum pepsinogen values. This study was conducted to evaluate the effect of a 7-year exposure to smoking, with special emphasis on whether or not the effect tends toward elevating risk of stomach cancer.

## SUBJECTS AND METHODS

Subjects were Japanese public service workers who participated in general health check programs in both 1989 and 1996. Ages of the subjects refer to their age in 1989. Serum pepsinogen values were measured using residual sera from the health check programs in both 1989 and 1996. Measurements were performed by BML Co. Ltd. (Tokyo) using the RIAbeads Pepsinogen I and II kits produced by Dainabot Co. Ltd. (Tokyo) in both 1989 and 1996 just after routine serum examinations of the health check programs. *Helicobacter pylori* antibody was measured by BML Co. Ltd. using residual sera from 1996. Pilika plate G Helicobacter II produced by Biomerica Co. Ltd. (Newport, CA) was used for the measurement. Instead of the kit-recommended cut-off value of 20.0 units/ml, the cut-off value was redefined as 16.0 units/ml, which yielded optimal sensitivity and specificity against the urea breath test among 492 Japanese subjects.^8^ The subjects were asked to fill out a questionnaire on smoking habits in both 1989 and 1996.

The subjects of the study were restricted to males, since there were so few female smokers. The subjects were classified into smoking and non-smoking groups: those who continued smoking and those who did not smoke during the 7-year span. Those who changed their smoking habit during the 7-year span and those with incomplete data were excluded from analyses. Serum PG I/II in 1989 and any changes during the 7-year span were compared between the non-smoking and the smoking groups. To observe changes in PG I/II, Δ PG I/II was defined as the PGI/II value in 1996 minus that in 1989. The mean of Δ PG I/II and the frequencies of subjects whose PG I/II increased during the 7-year span were calculated and compared between the non-smoking and the smoking groups. In the analyses, subjects were stratified by age (20-29 or 30-56 years; that is, 27-36 or 37-63 years in 1996) and *H. pylori* status (negative or positive).

Using multiple linear and logistic regression analyses, the age-adjusted effect of smoking on Δ PG I/II was calculated, among the *H. pylori* negative, positive and all subjects. In multiple linear regression analyses, the criterion variable was Δ PG I/II, and explanatory variables were age (years) and smoking habit (smoking or non-smoking during the 7-year span). In multiple logistic regression analyses, the criterion variable was whether Δ PG I/II was less than 0 or not, and the explanatory variables were the same as in the multiple linear regression analyses.

## RESULTS

**Figure 1.**
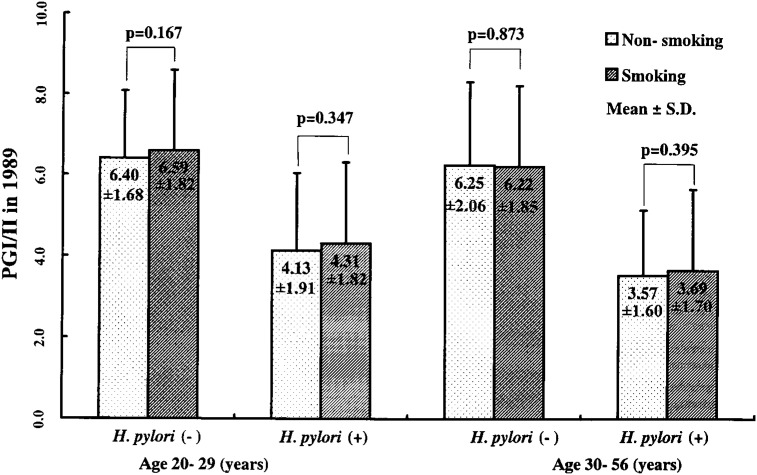
Mean ± standard deviation of pepsinogen I to II ratio (PG I/II) in 1989 in those without (Non-smoking) and in those with smoking habit (Smoking)

Among 2,336 male subjects, 256 were excluded from analyses because of incomplete information on their smoking habits. Age and smoking distribution of the subjects is shown in Table 1. Among the remaining 2,080 subjects, 191 who changed their smoking habit during the 7-year span were not included in analyses. Serum PG I/II values in 1989 are shown in Figure 1. Frequency of low PG I/II (i.e., less than 3.0) is shown in Table 2. *H. pylori*-negative subjects showed markedly higher PG I/II values in 1989 than *H. pylori*-positive subjects. On the other hand, no significant difference was observed between the smoking and the non-smoking group, although the smoking group showed slightly higher PG I/II values, except for *H. pylori*-negative subjects aged 30-56 years.

**Table 1.** Age and smoking habit of subjects

Age (years)*	Non-smoker		Inconsistent ¶		Smoker		Total	
20-29 (27-36)	396	( 37.9)	48	( 4.6)	600	( 57.5)	1044	(100.0)
30-39 (37-46)	290	( 33.8)	118	( 13.8)	450	( 52.4)	858	(100.0)
40-49 (47-56)	41	( 26.3)	21	( 13.5)	94	( 60.3)	156	(100.0)
50-56 (57-63)	5	( 23)	4	( 18)	13	( 59)	22	(100.0)
Total	732	( 35.2)	191	( 9.2)	1157	( 55.6)	2080	(100.0)

Number of subjects (%)

*In 1989 (in 1996)

¶ Those who changed their smoking habit during a 7-year span (Not included in the further analyses)

**Table 2.** Frequency of low pepsinogen I to II ratio in ’89

Age (years)	*H. pylori*	Non-smoker		Smoker	
20-29	Negative	7/241	( 2.9%)	14/380	( 3.7%)
	Positive	45/155	(29.0%)	44/220	(20.0%)
30-56	Negative	10/156	( 6.4%)	16/264	( 6.1%)
	Positive	77/180	(42.8%)	108/293	(36.9%)

Those whose PG I/II was less than 3.0 / number of subjects (percentage)

**Figure 2.**
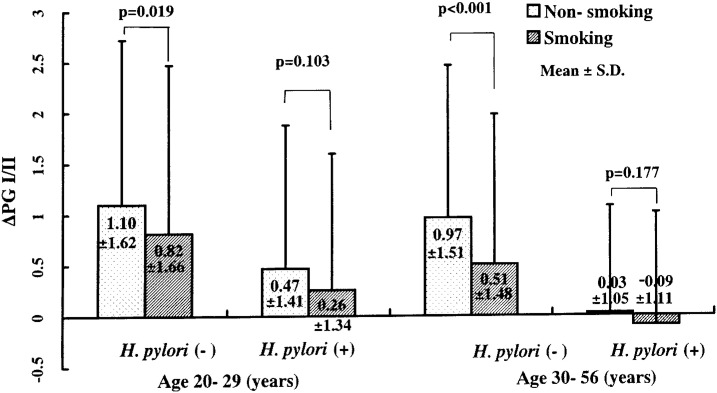
Mean ± standard deviations of Δ PG I/II (pepsinogen I to II ratio in 1996 minus that in 1989) during a 7-year span in those without (Non-smoking) and in those with smoking habit (Smoking)

**Figure 3.**
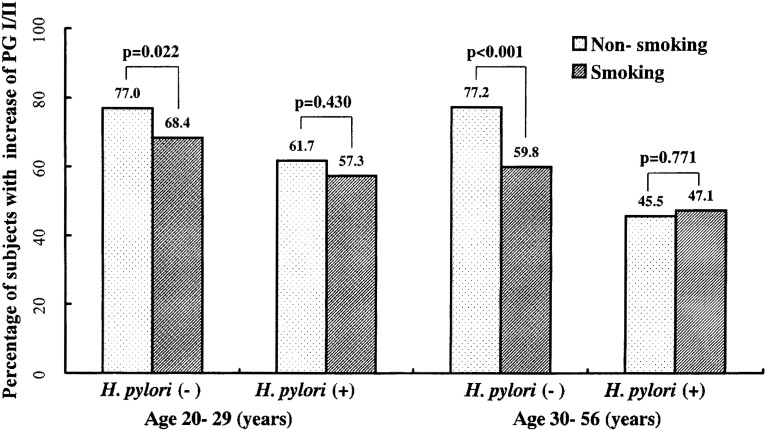
Percentage of subjects whose pepsinogen I to II ratio (PG I/II) increased during a 7-year span in those without (Non-smoking) and in those with smoking habit (Smoking)

Among *H. pylori*-negative subjects, the smoking group showed lower Δ PGI/II and less frequent increases in PG I/II than non-smoking group. Means ± standard deviations of Δ PGI/II among non-smokers and among smokers were 1.10 ± 1.62 and 0.82 ± 1.66 (p=0.02), respectively, in those aged 20-29 years, and 0.96 ± 1.51 and 0.51 ± 1.45, respectively (p<0.01), in those aged 30-56 years (Figures 2 and 3). In *H. pylori*-positive subjects, smokers tended to show lower Δ PG I/II and less frequent increases in PG I/II, but the results were not significant (Figures 2 and 3). In multiple regression analyses (Table 3), the smoking group showed significantly lower Δ PGI/II and less frequent increases in PG I/II than the non-smoking group among the *H. pylori*-negative and among all subjects. Among all the subjects, the mean ± standard error of the difference in Δ PGI/II adjusted for age was 0.21 ± 0.07 (p<0.01), and the frequency of increases in PG I/II was 65.8% in the non-smoking and 58.9% in the smoking groups (p<0.01).

**Table 3.** Difference in increase of serum pepsinogen I to II ratio (ΔPG I/II) and frequency of PG I/II increase during a 7-year span between non-smoking and smoking groups

Subjects	P.R.C. ± S.E.* of ΔPG I/II	p
Frequency of PG I/II increase (non-smoking versus smoking)		
*H. pylori negative*	0.339 ± 0.101	<0.001
	76.8% versus 64.9%	<0.001
*H. pylori positive*	0.072 ± 0.083	0.270
	52.8% versus 51.5%	0.805
All subjects	0.209 ± 0.069	0.002
	65.8% versus 58.9%	0.004

Adjusted for age using multiple linear/logistic regression analysis.

* Partial regression coefficient, that is mean of ΔPG I/II in non-smoking group minus that in smoking group. S.E.: standard error.

## DISCUSSION

### Mean, frequency and effect of age

There was no discrepancy between changes in PG I/II values and the frequency of subjects whose PG I/II values increased during the 7-year span, thus confirming the reliability of the results. Both facts are mentioned simply as changes in PG I/II values in the discussion. As PG I/II values are related with age,^9^ analyses were carried out with stratification by age or with age adjustments by multiple regression. Parallel results were obtained between the strata and when adjusted for age, which also enhances the reliability of the results.

During the 7-year span, the mean of PG I/II increased, whereas PG I/II has been negatively associated with age in several cross-sectional studies in Japan.^7^^,^^9^ There is an apparent inconsistency between the results of this study and those cross-sectional studies. Possible reasons for this inconsistency include a systemic error in measuring serum pepsinogen values, including deterioration of sera, a difference in the kits between 1989 and 1996, and bias in the measurements. However, the possibility of such bias is thought to be negligible, because sera were measured just after the routine examinations, and the measurements were performed by the same laboratory of one company in both 1989 and 1996 using the same kit. As pepsinogen I and II were measured in the same manner, the calculation of PG I/II may increase the stability of the results, counterbalancing systematic errors caused by such factors as individuality of the technician and temperature of the laboratory.

Another plausible explanation of the inconsistency is as follows. Few studies have so far observed changes in serum pepsinogens over a span of years. As shown in Figure 2, PG I/II increased among those aged 20-29 while it decreased among 30-56 in the *H. pylori*-positive subjects. On the other hand, it has been revealed that the prevalence of *H. pylori* in Japan is associated with factors in childhood such as sibship size and family history,^10^ and the prevalence depends more on birth year, which is strongly associated with sanitary conditions, than on mere biological age.^11^ Atrophy of the gastric mucosa depends more on *H. pylori* infection than on age.^12^ Therefore, it is suggested that the negative association between age and PG I/II in the cross-sectional studies is due to a cohort effect, and that the changes in PG I/II observed in this study reflect real relationship between biological age and PG I/II.

### Effect of *H. pylori*

*H. pylori* status was markedly associated with PG I/II values in 1989, and exerted a considerable effect on Δ PG I/II during the 7-year span,^13^ which is why the subjects were stratified by *H. pylori* status. Among the *H. pylori*-positive subjects, no significant difference in Δ PG I/II values was observed between the non-smoking and the smoking groups, although among the *H. pylori*-negative subjects there were significant differences in Δ PG I/II values. *H. pylori* seems to have exerted a stronger effect than smoking. Actually, PG I/II values and their changes during the 7-year span depended more on the *H. pylori* status than on smoking.

### Effect of smoking on serum pepsinogen

In 1989, the smoking group showed slightly higher PG I/II values than the non-smoking group except for *H. pylori*-negative subjects aged 30-56 years, but the differences were not significant. Such results were consistent with the results of a Japanese study^14^ which concluded that smoking had no significant effect on PG I/II values when stratified by *H. pylori* status. Our previous study showed a positive association between current smoking levels and PG I/II values.^7^ This inconsistency may be because our previous study was without stratification by *H. pylori* status, and because our previous study using a large sample size detected small differences in PG I/II values brought about by smoking as significant difference.

In *H. pylori*-negative subjects, the smoking group showed significantly lower Δ PG I/II than the non-smoking group. In *H. pylori*-positive subjects, the difference between the two groups was not significant, although the smoking group also showed lower Δ PG I/II. The effect of smoking on PG I/II was clear in *H. pylori*-negative subjects, but not in *H. pylori*-positive subjects. The ambiguous effect of smoking on *H. pylori*-positive subjects may be partly due to low PG I/II values in 1989 compared with *H. pylori*-negative subjects. There was no significant difference in PG I/II values in 1989 between the smoking and the non-smoking groups in any strata by age or *H. pylori* status. Among those aged 20-29 years, gaps in PG I/II values in 1989 between the smoking and the non-smoking groups were almost the same between *H. pylori*-negative and positive subjects. However, the effect of smoking on Δ PG I/II was significant only in *H. pylori*-negative subjects. Differences in PG I/II in 1989 between the smoking and the non-smoking groups seemed to have no association with Δ PG I/II. It is concluded that the less frequent increase (i.e., more frequent decline) of PG I/II is a long-term effect of smoking on serum pepsinogens, although the effect is not clear under *H. pylori* infection.

PG I, and consequently PG I/II, are known to reflect atrophy of the gastric mucosa.^15^^-^^17^ Recent studies have revealed that PG I/II reflects both atrophy and inflammation of the gastric mucosa, but Δ PG I/II is thought to reflect mainly the advance of atrophy because the influence of inflammation is counterbalanced in the calculation.^13^ The frequent decline in PG I/II in the smoking group may reflect the advance of gastric mucosal atrophy. Because nicotine in tobacco smoke stimulates secretion of pepsinogens,^18^ smoking elevates the level of pepsinogen I^7^^,^^14^ but not pepsinogen II in the short-term.^7^ However, cigarette-year (the total amount a subject had ever smoked) was negatively related with pepsinogen I but not related with pepsinogen II,^7^ i.e., smoking brought about a decline in pepsinogen I and a subsequent decline in PG I/II over the long-term. The long-term effect of smoking may be because long-term stimulation of pepsinogen secretion causes the exhaustion of chief cells. These results were consistent with the results of the current study. Smoking may stimulate pepsinogen secretion, and such long-term stimulation may exhaust the chief cells and promote atrophy of the gastric mucosa.

### Smoking and stomach cancer

Smoking is a suspected risk factor for stomach cancer, although this is equivocal.^4^^,^^19^^-^^26^ In the current study, smoking brought about a frequent decline in PGI/II during the 7-year span. As discussed above, low PG I/II is closely related to atrophy of the gastric mucosa and chronic atrophic gastritis,^27^ which in tern is a precursor of stomach cancer.^28^^,^^29^ A direct strong association between low PG I/II and the risk of stomach cancer has been shown.^1^^-^^3^^,^^30^ It is, therefore, expected that smoking may elevate the risk of stomach cancer by provoking a decline in PG I/II, although its effect is weaker than that of *H. pylori*.
